# Raynaud's Phenomenon

**Published:** 2013-09-10

**Authors:** Jehan Shah, Alicia R. Billington, Joshua B. Elston, Wyatt G. Payne

**Affiliations:** Plastic Surgery Section, Bay Pines VA Healthcare System, Bay Pines, Fla; and the Division of Plastic Surgery, Department of Surgery, University of South Florida College of Medicine, Tampa, Fla

**Keywords:** Raynaud's, sympathectomy, systemic sclerosis, angiography, digital ischemia

## DESCRIPTION

**Figure 1:**
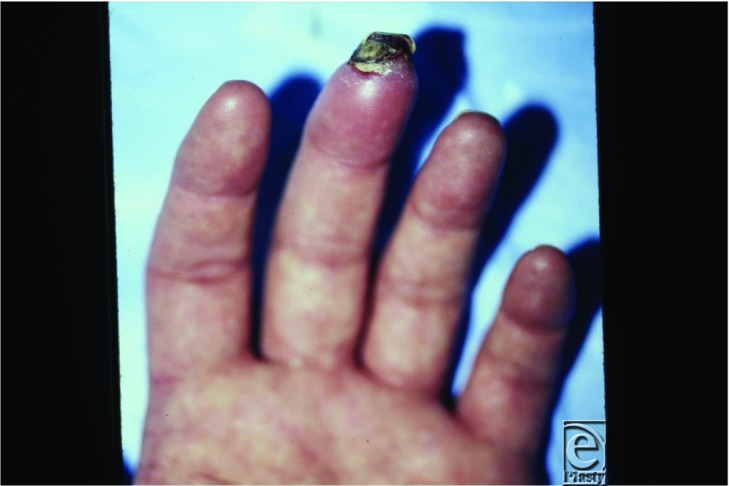
Ischemic ulceration and necrosis of left middle finger.

A 35-year-old man presented with a 3-year history of episodic fingertip pain with associated discoloration triggered by cold exposure. His job required activities in a subzero walk-in freezer, which exacerbated pain and discoloration. The middle fingertip wound began spontaneously 3 months prior and worsened despite medical therapy (Fig 1).

## QUESTIONS

1. **What is the difference between primary and secondary Raynaud's?**
2. **What is the pathophysiology of Raynaud's?**
3. **What evaluation options are available for diagnosis of Raynaud's?**
4. **What are the management options?**

## DISCUSSION

Raynaud's phenomenon (RP) is characterized by episodic vasospasms of the digits upon exposure to cold or psychosocial stressors that result in sequential color changes: white to blue to red. These color changes correspond to ischemia, deoxygenation, and hyperemia, respectively. When vasospasm is associated with a pathologic etiology such as systemic sclerosis, the diagnosis of secondary RP or Raynaud's syndrome is appropriate. Primary RP is known as Raynaud's disease once the secondary causes have been ruled out. Determining the appropriate diagnosis becomes important as vasospasms can predate the onset of an autoimmune disorder by as early as 20 years.^1^

The elusive pathophysiology of RP seems to be intricately linked to chemical and anatomic intravascular factors as well as neural modulators. Decreased production of nitric oxide decreases vasodilation and increased production of endothelin-1 (a potent vasoconstrictor) causes a hyperbolic arterial constriction.^2^ Anatomic abnormalities, such as intimal thickening possibly mediated by upregulated profibrotic angiotensin and endothelin-1, lead to increased intravascular resistance and provide an environment whereby flow is limited similar to peripheral arterial disease.^2^^,^^3^ Platelet activation releases thromboxane A^2^ causing further vasoconstriction, platelet aggregation, and primary clot formation. This confluence of events ultimately results in ischemia-reperfusion injury or in the worst case, arterial thrombosis.

The work-up of patients with suspected RP includes a CBC to rule out malignancy and polycythemic syndromes, autoimmune antibody panels, and a hepatitis panel for cryoglobulinemia. Diagnosis of RP is suggested if rewarming takes more than 20 minutes after performance of cold stress test. Nailfold videocapillaroscopy is an optical microscopic technique that allows visual evaluation of nailfold microvasculature and is considered the gold standard for diagnosis of RP.^1^ Pathologic findings such as microhemorrhages, giant capillaries, and avascular regions are some of the earliest diagnostic signs of systemic sclerosis.^4^

If conservative measures (placing hand in warm water, shaking hand to force blood distally) during an acute vasospastic episode do not relieve symptoms, calcium channel blockers such as nifedipine or diltiazem are considered a first-line treatment for uncomplicated RP. Phosphodiesterase V inhibitors are considered to be second-line treatment and when combined with bosentan have been shown to treat and prevent ulcers, especially in patients with complicated secondary RP due to systemic sclerosis.^5^ Botulinum toxin A injection of the hand has also been utilized for symptomatic relief with good results. Proposed mechanisms of action include increased blood flow by inhibition of sympathetic vasoconstriction and decreased pain perception due to decreased activity of C-fiber nociceptors.^6^ Surgical intervention is indicated in patients who are refractory to medical therapy or with infected ulcers/necrotic digits. Angiography is important to determine the appropriate level and type of intervention as it provides a surgical roadmap of the affected vessels. One recent publication proposed a classification system and interventions (vessel grafting, sympathectomy, or balloon angioplasty) for RP based on angiographic findings.^7^

**Figure 2:**
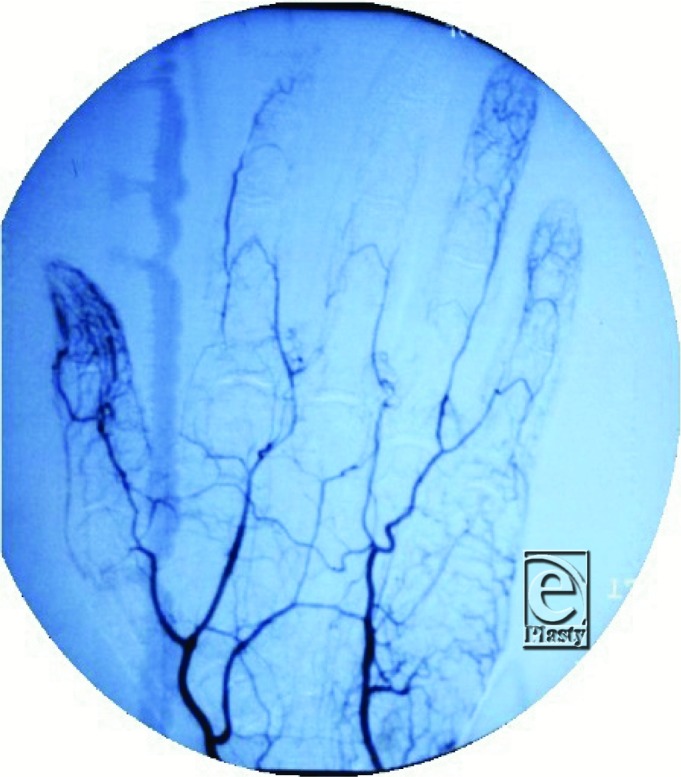
Preoperative angiogram revealing moderately narrowed ulnar artery with vasospastic palmar arch vessels and digital vessels.

The incidence of RP is rare, but female patients tend to present earlier with a female:male ratio of 4:1. Vibratory sensations, such as catching a baseball or working on industrial machinery, are also known to provoke an episode of vasospasm in addition to the commonly cited inducers, cold and stress. While primary Raynaud's may cause pain, that is usually the extent of symptomatology as any ulceration precludes this diagnosis and should prompt evaluation for underlying systemic diseases as RP can herald a more diffuse and severe process.^1^ Our patient presented with RP associated with a moderately narrowed ulnar artery, severely narrowed palmar arch, and trickle flow to the common digital artery (Fig 2).

**Figure 3:**
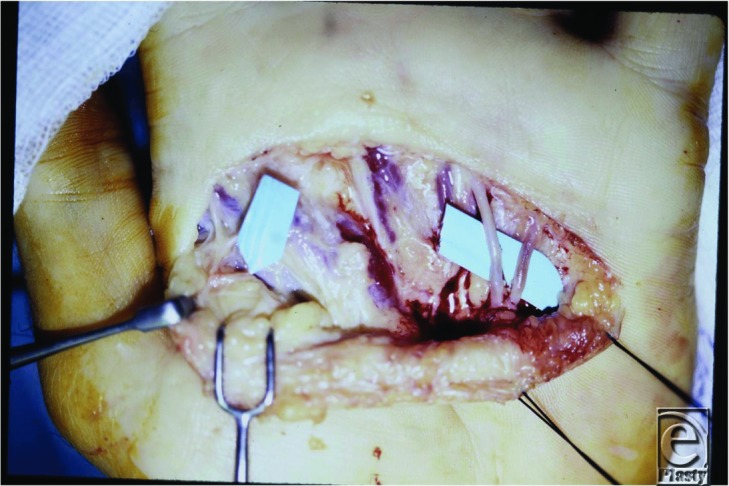
Intraoperative photo. Note the increased ulnar-sided vessel caliber postsympathectomy relative to the radial-sided unoperated vessels.

**Figure 4:**
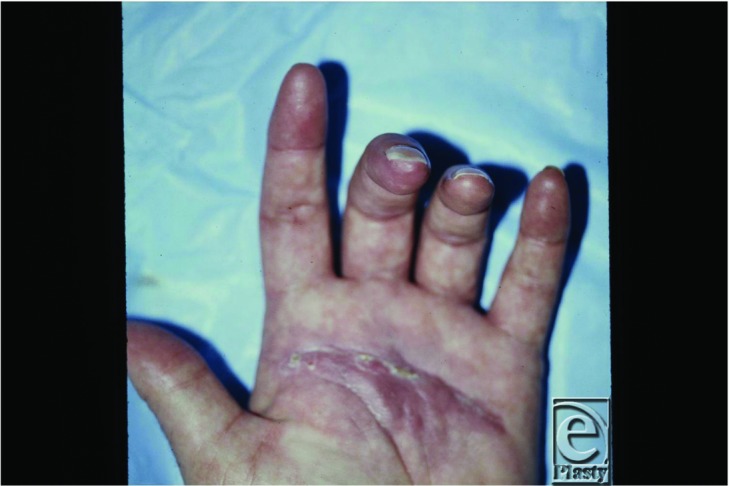
Two months postoperatively with healing of the digit.

A digital sympathectomy provided immediate dilation of the stenotic vessels (Fig 3) and greatly reduced symptoms at 2-month follow-up with resolution of fingertip ulceration (Fig 4).
